# Survey of understanding and awareness of fertility preservation in pediatric patients: Is conversation about fertility preservation unpleasant for pediatric patients?

**DOI:** 10.3389/fendo.2022.1074603

**Published:** 2023-01-06

**Authors:** Seido Takae, Yuriko Iwahata, Yodo Sugishita, Hideyuki Iwahata, Ryo Kanamori, Eriko Shiraishi, Kaoru Ito, Yuki Suzuki, Yoshiko Yamaya, Kunihide Tanaka, Kei Oyama, Dai Keino, Kentaro Nakamura, Kei Odawara, Yuki Horage, Lingbo Meng, Arby Igualada, Ahmad Mohd Faizal, Ludmilla Ogouma Aworet, Shigeyuki Furuta, Miki Sakamoto, Tetsuya Mori, Hiroaki Kitagawa, Nao Suzuki

**Affiliations:** ^1^ Department of Obstetrics and Gynecology, St. Marianna University School of Medicine, Kawasaki, Kanagawa, Japan; ^2^ Department of Obstetrics and Gynecology, The Jikei University School of Medicine, Tokyo, Japan; ^3^ Department of Pediatric Surgery, St. Marianna University School of Medicine, Kawasaki, Japan; ^4^ Department of Hematology and Oncology Pediatric Department Kanagawa Children’s Medical Center, Yokohama, Kanagawa, Japan; ^5^ Department of Pediatrics, St. Marianna University School of Medicine, Kawasaki, Kanagawa, Japan; ^6^ Department of ObGyn, UKM Medical Centre, Cheras, Kuala Lumpur, Malaysia; ^7^ Department of Anesthesiology, St. Marianna University School of Medicine, Kawasaki, Kanagawa, Japan

**Keywords:** fertility preservation, pediatric cancer, ovarian tissue cryopreservation, oocyte cryopreservation, oncofertility, children’s response to fertility preservation explanations

## Abstract

**Objective:**

To verify understanding and awareness of fertility preservation (FP) in pediatric patients undergoing FP treatments.

**Methods:**

A questionnaire survey was conducted before and after explanation of fertility issues and FP treatments for patients 6–17 years old who visited or were hospitalized for the purpose of ovarian tissue cryopreservation (OTC) or oocyte cryopreservation (OC), or sperm cryopreservation between October 2018 and April 2022. This study was approved by the institutional review board at St. Marianna University School of Medicine (No. 4123, UMIN000046125).

**Result:**

Participants in the study comprised 36 children (34 girls, 2 boys). Overall mean age was 13.3 ± 3.0 years. The underlying diseases were diverse, with leukemia in 14 patients (38.9%), brain tumor in 4 patients (11.1%). The questionnaire survey before the explanation showed that 19 patients (52.8%) wanted to have children in the future, but 15 (41.7%) were unsure of future wishes to raise children. And most children expressed some degree of understanding of the treatment being planned for the underlying disease (34, 94.4%). Similarly, most children understood that the treatment would affect their fertility (33, 91.7%). When asked if they would like to hear a story about how to become a mother or father after FP which including information of FP, half answered “Don’t mind” (18, 50.0%). After being provided with information about FP treatment, all participants answered that they understood the adverse effects on fertility of treatments for the underlying disease. Regarding FP treatment, 32 children (88.9%) expressed understanding for FP and 26 (72.2%) wished to receive FP. “Fear” and “Pain” and “Costs” were frequently cited as concerns about FP. Following explanations, 33 children (91.7%) answered “Happy I heard the story” and no children answered, “Wish I hadn’t heard the story”. Finally, 28 of the 34 girls (82.4%) underwent OTC and one girl underwent OC.

**Discussion:**

The fact that all patients responded positively to the explanations of FP treatment is very informative. This is considered largely attributable to the patients themselves being involved in the decision-making process for FP.

**Conclusions:**

Explanations of FP for children appear valid if age-appropriate explanations are provided.

## 1 Introduction

In recent years, interest in post-treatment quality of life and the late complications of cancer survivors has been increasing. Furthermore, with the aim of popularizing and developing fertility preservation (FP), the “Oncofertility Consortium” and “FertiPROTEKT” were established in 2006, followed by the International Society for Fertility Preservation in 2009, the Japan Society for Fertility Preservation (JSFP) in 2012, and the Asian Society for Fertility Preservation in 2015 (1). The importance of FP has also been recognized more widely in society, and public financial support for FP became available in Japan in 2021. The number of patients desiring FP is thus expected to increase in the future. In particular, the number of cases involving prepubertal children, for whom FP has been difficult to implement, seems likely to increase markedly. Further, major organizations such as the American Society of Clinical Oncology (2) (3) (4),,, European Society of Medical Oncology (5), and European Society of Human Reproduction and Embryology (6) have all published FP guidelines, and in Japan, the Japan Society of Clinical Oncology and JSFP have jointly published FP guidelines (7) and were revising these guidelines as of October 2022. These advanced guidelines have eliminated lower age limits, but do not provide specific methods for implementing FP in infants and prepubertal children (8). This is understandable, since FP initially continued to develop technologically for adults such as breast cancer patients. However, since pediatric patients with blood diseases are increasingly major targets of FP measures, clarifying specific methods of dealing with pediatric patients is crucial. In fact, the spread of FP to pediatric patients is reportedly delayed in Asia compared to adult patients, due to difficulties with providing information, a lack of explanatory materials, and lack of cooperation with pediatricians (1). In addition, when a child receives information about FP in the first place, the intention of the parent has a substantial influence, and the parent may act as a barrier and not provide information to the child. Unsurprisingly, parents consider various factors such as the age of the child, the degree of intellectual development, context and timeline for decision-making, costs, the mental health of the child, the invasiveness of potential FP interventions, and culture. At some point, medical staff may finally arrive at the point of providing information on FP to the child (9). Few clinical studies with comparisons to adults have been conducted, and no reports have described the psychological problems and comprehension of pediatric patients themselves due to the difficulties inherent in conducting such investigations.

We therefore undertook a preliminary survey on the perception of FP in pediatric patients, the degree of understanding of FP after provision of an explanation, and feelings toward FP. We believe that this survey will provide basic insights into the actual feelings of pediatric patients and should contribute to the implementation of high-quality FP for pediatric patients.

## 2 Materials and methods

### 2.1 Patients

Participants in this study were children between 6 and 17 years old who visited our hospital for the purposes of FP consultation or who were hospitalized for the purpose of OTC between October 2018 and July 2022. All children had a malignant disease such as leukemia or a disease such as aplastic anemia or chronic active Epstein-Barr virus (EBV) infection, in which there was a possibility that treatment of the primary disease with chemotherapy and/or radiotherapy would greatly impair future fertility.

### 2.2 Explanation of FP

Before providing an explanation of FP, we gave the parents and child a leaflet created by the JSFP. After reading the leaflet, the parents and child were given an explanation about FP alike telling a story (suppl. 1). However, we did not verify whether parents had told the child about the contents of the leaflet before the explanation. During the actual explanation, a male FP doctor specializing in reproductive medicine (including FP) and endoscopic surgery subjectively evaluated the development status of child while talking with the child and explaining FP by drawing pictures on a piece of paper. So, the explanation of the FP seemed to tell the story. Moreover, it was remarkable when explaining to younger children. Also, he and young female assistant doctors also provided explanation using an original animated movie currently under development as a supplement. The method of explanation was changed taking into consideration not only age but also comprehension. Basically, after having girls in their teens and beyond puberty read the leaflet, we explained it including a certain degree of specialized knowledge. For elementary school students, we asked their parents to explain without reading the leaflet, and for younger children, we mainly explained with parable. When the subject of the explanation for FP was a girl, consideration was given to female doctors and nurses in attendance as much as possible, so that the explanation would not be given only by male doctors.

### 2.3 Questionnaire survey

Before and after the explanation of FP, a questionnaire survey was conducted to evaluate the feelings and perceptions of the child. There was no age-specific version of the survey, and for children who could not read or write sufficiently, parents explained the content and filled it out. Children who can read and write on their own were basically included while confirming the content with their parents. Most of the children after puberty checked the contents by themselves and answered by themselves. Therefore, it cannot be denied that the reliability of this survey declines with low age. The contents of questions before the explanation of FP consisted of 10 questions, including the sex and age of the child. The contents were “Do you know about the planned treatment for your illness?”, “Do you know that treating your illness may make it harder for you to become a mother or father in the future?”, and so on. Details of the questions before providing the explanation about FP are shown in **Table 1**. The number of questions posed after the explanation about FP was smaller than before the explanation, in consideration of the physical and mental burden of the child. There were 8 questions, with the contents designed to elucidate changes in knowledge, such as “Do you understand the effect of treatment for your illness on your chances of being a mother or father in the future?”, and “Do you understand the medical technology to preserve your chances of being a mother or father in the future?”, and so on. In addition, questions such as “Do you want to receive medical technology to preserve your chances of being a mother or father in the future?”, and “After these explanations, have you noticed any change in your desire to become a mother or father?”, were posed. Questions asking about the psychological state of the child, such as “Please tell us how you felt after listening to the story.” were also set. Details of the questions after the explanation about FP are shown in **Table 2**. The questionnaires used in this study were evaluated and modified by the researchers and psychologists specializing in reproductive medicine and FP.

**Table 1:** Questionnaire pre-explanation of fertility preservation.

Pre	Questions	Answers
Q1	Please tell me your gender.	(boy, girl)
Q2	How old are you?	() years old
Q3	Do you want to have your own children when you grow up? (Would you like to be a mother or father)?	(I really think so, I think so, I don’t know yet, I don’t think so, I really don’t think so)
Q4	Do you know about the planned treatment for your illness?	(Know well, Know, Know a little, Don’t really know, Don’t know anything)
Q5*	Please indicate what you know about treatment for Q4.	(Surgery, Chemotherapy (drugs), Radiotherapy, Hematopoietic cell transplantation, Other, Don’t know)
Q6	Do you know that treating your illness may make it harder for you to become a mother or father in the future?	(Know well, Know, Know a little, Don’t really know, Don’t know anything)
Q7	Do you know what you came to hear?	(Know well, Know, Know a little, Don’t really know, Don’t know anything)
Q8	Would you like to hear a story about how to become a mother or father in the future (after FP)?	(Really want to hear, Want to hear, Don’t mind, Don’t really want to hear, Don’t want to hear at all)
Q9	Do you know that there are medical technologies that can help you become a mother or father in the future?	(Know well, Know, Know a little, Don’t really know, Don’t know anything)
Q10*	Please indicate what treatments you know for Q9.	(OC, OTC, Ovarian suppression, Sperm cryopreservation, Testicular tissue cryopreservation, Gonadal shielding, Don’t know)
		*Multiple selections allowed

Before providing an explanation about fertility preservation, knowledge about the treatment for the underlying disease of the child and knowledge about fertility preservation were examined.

OC, oocyte cryopreservation; OTC, ovarian tissue cryopreservation; FP, fertility preservation.

**Table 2:** Questionnaire post-explanation of fertility preservation.

Post	Questions	Answers
Q1	Do you understand the effect of treatment for your illness on your chances of being a mother or father in the future?	(Understand well, Understand, Understand a little, Don’t really understand, Don’t understand at all)
Q2	Do you understand the medical technology to preserve your chances of being a mother or father in the future?	(Understand well, Understand, Understand a little, Don’t really understand, Don’t understand at all)
Q3	Do you want to receive medical technology to preserve your chances of being a mother or father in the future?	(Really want to receive FP treatment, Maybe want to receive FP treatment, Don’t know, Don’t really want to receive FP treatment, Don’t want to receive FP treatment at all)
Q4	Please indicate the medical technologies you may receive.	(OC, OTC, Ovarian suppression with GnRH agonist, Sperm cryopreservation, Testicular tissue cryopreservation, Gonadal shielding against radiation, Don’t really understand)
Q5*	What are your concerns about receiving the treatment you selected in Q4?	(Fear, Worried about pain, Not sure what to do, Worried about costs, Don’t want to be transferred, Don’t understand the need for FP, Don’t want to receive any more burdensome treatments, Worried but unsure why)
Q6	After these explanations, have you noticed any change in your desire to become a mother or father?	(Yes, No, Don’t know)
Q7	If there were any changes in Q6, please tell us what they are.	(Greater desire to have children, Greater desire to not have children, Other)
Q8	Please tell us how you felt after listening to the story.	(Happy I heard the story, Wish I hadn’t heard the story, Didn’t really understand, Other)
		*Multiple selections allowed

After receiving the explanation about fertility preservation, the reactions, concerns, and impressions of the child to the explanation were examined.

OC, oocyte cryopreservation; OTC, ovarian tissue cryopreservation; FP, fertility preservation, GnRH, gonadotropin-releasing hormone.

### 2.4 Ethical considerations

Since the content of the questions set in this study was very sensitive, the questionnaire was filled out with the parents after guaranteeing the right to refuse to answer at any time. In addition, the questionnaire was conducted under careful observation by the medical staff to see if the child exhibited any physical or mental changes while answering the questionnaire. This study was conducted under the approval of the Institutional Review Board at St. Marianna University School of Medicine (approval no. 4123, UMIN000046125).

## 3 Results

### 3.1 Characteristics of patients

**Figure 1:**
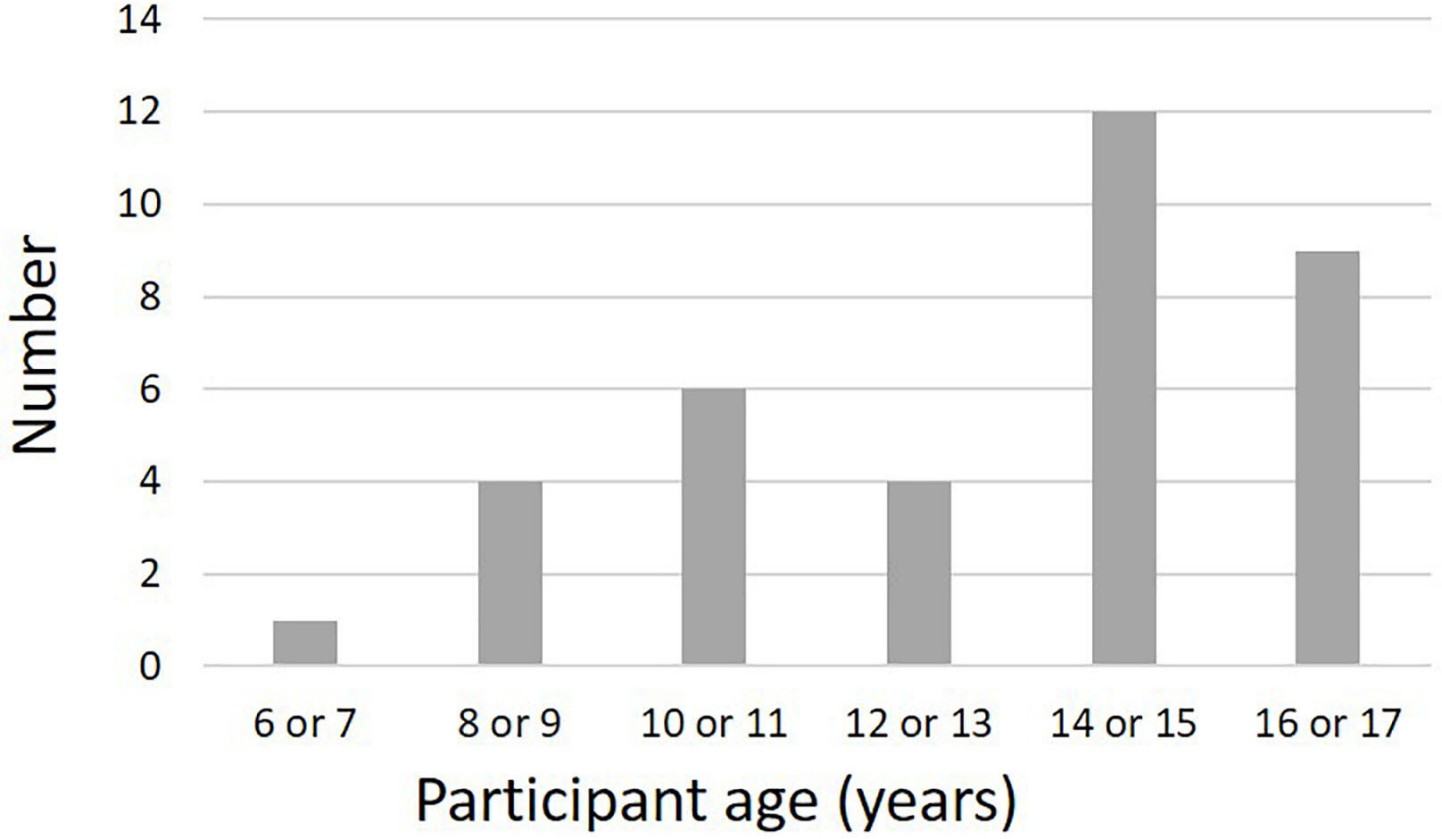
Age distribution of participants in the present study. The peak age groups were 14–15 years and 16–17 years. Participants also included 11 prepubertal children ≤11 years old. The youngest participant was 6 years old.

A total of 36 pediatric patients participated in this study, with a response rate of 100%. All children and parents agreed to participate in this study. Thirty-four of the 36 patients were girls and the other 2 were boys. The overall median age was 13.3 ± 3.0 years old. The median age of girls was 14 years (range, 6–17 years). The boys were 14 and 15 years old and the primary illness in both cases was ALL. Of these 36 patients, 11 were <11 years old (Group A), 9 were 12–14 years old (Group B), and 16 were 15–17 years old (Group C). **Figure 1** shows the age distribution of study participants. In addition, the primary diseases of girls were diverse, with 14 leukemias (38.9%), 4 brain tumors (11.1%), 4 rhabdomyosarcomas (11.1%), 4 other sarcomas (11.1%, such as Ewing’s sarcoma and osteosarcoma), and 3 malignant lymphomas (8.3%). In addition, anaplastic anemia was present in 2 children, and myelodysplastic syndrome (MDS), mediastinal tumors, systemic lupus erythematosus (SLE), chronic active Epstein-Barr virus infection (CAEBV), and thalassemia in 1 patient each. Most were primary cases, but two patients (including one boy) were relapse cases. In addition, 25 patients (69.4%) were already undergoing chemotherapy at the time of their visit, and most of the remaining patients had already undergone surgery, immunosuppressant therapy, blood transfusion therapy, etc.; only one patient was completely untreated.

As a result of the explanation about FP, two boys chose sperm cryopreservation, but one was already suffering chemotherapy-induced azoospermia. In addition, 28 girls (82.4%) underwent OTC and one underwent OC, whereas two girls thought that treatment of the underlying disease would have no significant effect on their own ovarian reserve and decided to follow-up on ovarian reserve. Notably, one girl initially chose OTC and was hospitalized, but her feelings changed immediately before the procedure and she eventually declined to undergo OTC. In addition, two girls prioritized treatment of the underlying disease and did not choose any FP. Generally, at our institution, OTC is performed by single-port laparoscopic surgery, and the most important thing is to perform minimally invasive and safe procedures based on the policy of reduced-port surgery.

### 3.2 Questionnaire survey results before explanation about FP

**Figure 2:**
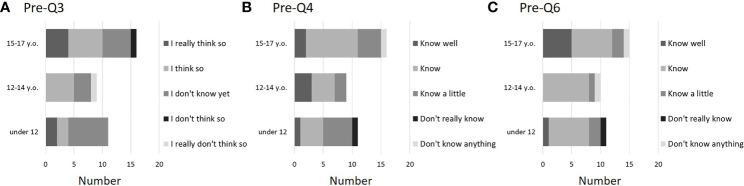
Questionnaire results before FP explanation (pre-Q3, 4, and 6). About half of the affected children expressed hopes of becoming parents in the future, but some (especially young children) were unsure of their feelings about “wanting to become father or mother” (**A**: pre-Q3). In addition, some children (particularly among young children) did not understand the planned treatment (**B**: pre-Q4). On the other hand, children tended to gain an understanding of the effects of disease treatment on fertility and the reasons for their visit (**C**: pre-Q6). Of these 36 patients, 11 were <11 years old (Group A), 9 were 12–14 years old (Group B), and 16 were 15–17 years old (Group C).

**Figure 3:**
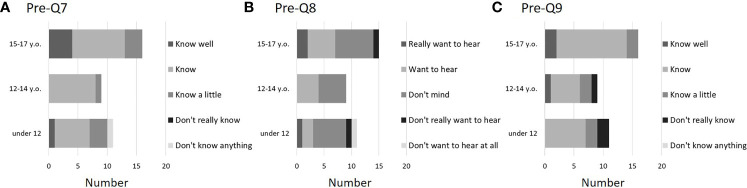
Questionnaire results before FP explanation (pre-Q7, 8, and 9). Similar to pre-Q6, many children recognized the reasons for visiting our hospital (**A**: pre-Q7). However, not many children were enthusiastic about hearing a story about becoming a parent in the future, consistent with Q3 (**B**: pre-Q8). In addition, many children were aware of FP treatment, which was generally consistent with pre-Q6 and -Q7(**C**: pre-Q9). Of these 36 patients, 11 were <12 years old (Group A), 9 were 12–14 years old (Group B), and 16 were 15–17 years old (Group C).

All children were able to answer about their age and biological sex (Q1 and 2). Regarding Q3 (desire to have children in the future), more than half of Groups B and C answered, “I really think so” or “I think so”. However, although no negative answers were seen in the younger Group A, 7 children (63.6%) answered that “I don’t know yet” (**Figure 2A**). Regarding Q4, in Groups B and C, patients knew the treatment for the underlying disease that they were planning to receive, but in Group A, 5 of the 11 respondents (45.5%) said they “Know a little”. A small number of children were unaware of the treatment being planned (one patient each in Groups A and C) (**Figure 2B**). Q5 asked for details about the subsequent planned treatment. Of the 36 patients, 17 (47.2%) responded that the treatment would be surgical, 21 (58.3%) responded chemotherapy, 12 (33.3%) responded radiation therapy, and 8 (22.2%) responded hematopoietic cell transplantation (multiple answers allowed). In addition, most children understood that treatment for the underlying disease would adversely affect fertility (Q6), with 8 of 11 (72.2%) in younger Group A, 8 of 9 (88.9%) in Group B, and 12 of 16 (75%) in Group C answering that they “Know well” or “Know”. In Group C, which is the upper grades, the percentage of those answering “Know well” was high, at 5 of 16 children (31.2%). One child in each age group answered “Don’t really know” or “Don’t know anything” (**Figure 2C**). In Q7, which asked about the purpose of coming to our hospital, most children answered that they “Know well”, “Know”, or “Know a little” (**Figure 3A**). Regarding Q8, which asked whether they would like to hear explanation about the process from FP to assisted reproductive medicine, less than half of the children answered “Really want to hear” or “Want to hear”, and many answered “Don’t mind” (**Figure 3B**). This tendency was particularly noticeable in Groups A and B, who were younger, with 6 of 11 (54.5%) children in Group A and 5 of 9 (55.6%) children in Group B responding “Don’t mind”. Many children were aware of the existence of FP treatment (Q9), especially among the older children in Group C, and all were at least somewhat aware (**Figure 3C**). Regarding Q10, the most common FP treatments they knew of were OC (21 of 36, 58.3%) and OTC (18 of 36, 50.0%). Five patients were also aware of sperm cryopreservation (13.9%), including one boy, and three were aware of testicular tissue cryopreservation. All 4 girls who knew about sperm cryopreservation and all 3 girls who knew about testicular tissue cryopreservation were Group C girls. Regarding medical terminology, were given to the children by medical staffs or parents as appropriate. Therefore, there were no major difficulties in conducting present clinical studies

### 3.3 Questionnaire survey results after explanation about FP

**Figure 4:**
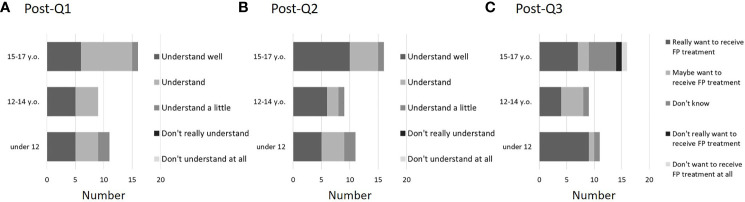
Questionnaire results after FP explanation (post-Q1, 2, and 3). A certain degree of understanding was reported, and no patients reported no understanding at all of the effects of treatment on fertility or of FP treatment (**A**: post-Q1; **B**: post-Q2). In addition, two participants aged 15–17 answered that they did not want to undergo FP, but one (a boy) subsequently decided to undergo sperm cryopreservation. The other (a girl) did not meet the indications for FP (**C**: post-Q3). Of these 36 patients, 11 were <11 years old (Group A), 9 were 12–14 years old (Group B), and 16 were 15–17 years old (Group C).

**Figure 5:**
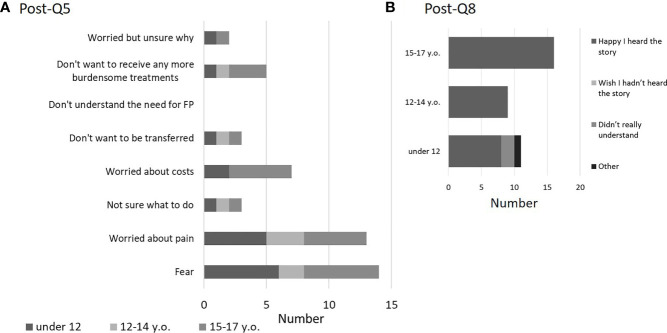
Questionnaire results after FP explanation (post-Q5 and 8). Key concerns about FP involved fear and pain, followed by financial concerns. No age-related differences were seen in these results (**A**: post-Q5). After the explanation, all children showed a positive reaction. The results also included patients (5 girls) who did not receive FP and the patients (one girl and boy) who responded that they did not wish to undergo FP (**B**: post- Q8). Of these 36 patients, 11 were <11 years old (Group A), 9 were 12–14 years old (Group B), and 16 were 15–17 years old (Group C).

After the explanation, most children understood the effects on fertility of the treatment for the underlying disease (post-Q1). None of the children answered, “Don’t really understand” or “Don’t understand at all” (**Figure 4A**). Regarding FP treatment, everyone answered that they understood to some degree, including “Understand a little” (post-Q2) (**Figure 4B**). When asked if they would like to receive FP treatment (post-Q3), 10 of 11 children (90.9%) in Group A, 8 of 9 children (88.9%) in Group B, and 9 of 16 (56.3%) in Group C answered they “Really want to receive FP treatment” or “Maybe want to receive FP treatment”. In addition, a girl who answered that “Don’t want to receive FP treatment at all” was not indicated for FP treatment because the treatment for the primary disease had not so large effect on fertility. A boy who answered “Don’t really want to receive FP treatment” underwent sperm cryopreservation (**Figure 4C**). Regarding FP treatments that they may receive (post-Q4), 7 of 36 children (19.4%) responded “OC”, 30 of 36 (83.3%) responded “OTC”, 2 responded “Sperm cryopreservation”, and 2 responded “Ovarian suppression with GnRH agonist”. In addition, no children answered, “Testicular tissue cryopreservation” or “Gonadal shielding against radiation”, but 3 of 36 (8.3%) responded that “Don’t really understand” (2 in Group A and 1 in Group C). Post-Q5 asked about anxiety factors related to FP treatment. Ten of the 36 children (27.8%) did not answer this question (**Figure 5A**). The most common anxiety factor was “Fear” (14 of 36, 38.9%), followed by “Worried about pain” (13 of 36, 36.1%). The next most common answer was “Worried about costs”. Two of 11 respondents (18.1%) in Group A and 5 of 16 (31.3%) in Group C answered “Worried about costs”. After explaining about FP, only 3 children reported a change in their desire to have children, with a positive change in 2 of the 3 children, and no response in the remaining (post-Q6 and 7). Finally, in post-Q8, which asked about impressions of the explanations for FP, 33 of the 36 children (91.7%) answered “Happy I heard the story” and 2 children (6- and 11-year-old girls) from Group A answered, “Didn’t really understand”. None of the children answered, “Wish I hadn’t heard the story” (**Figure 5B**).

## 4 Discussion

FP treatment for pediatric patients has gained popularity in recent years, and the number of cases has been increasing (10–14). This trend is spreading not only in Europe and the United States, but also throughout Asia and worldwide (15, 16). In addition, most reports have been on ovarian tissue freezing in pediatric FP, with only a few reports on OC or sperm freezing, as was the case in this study (17). Pediatric FP, whether OTC or OC, shows many scientific and medical differences from adult FP. The scientific aspects have been partially clarified with the gradual accumulation of knowledge about FP in children, but many years will be required to demonstrate its efficacy. Regarding medical care, pediatric patients reportedly often have systemic diseases, making OTC difficult to implement. Therefore, risk management at the time of surgery, ingenuity in surgical techniques, and cooperation among clinical departments have been shown to be necessary for success (18, 19). Particular attention should also be paid to ethical considerations, and due consideration of psychological issues is extremely important (14, 20). This study focused on and investigated psychological aspects of decision making for children considering FP, because the stress and fear of considering FP while battling a disease is likely to be much greater than that in adults. This is compounded by a lack of understanding or misunderstanding of FP due to the young age of the patients, underlying disease, and inadequate explanations from medical staff.

The results of this study showed that even minors who have not faced pregnancy, childbirth, or marriage have a strong desire to have children in the future (Q3). However, the lower the age, the more often the answer was “I don’t know yet”. Previous studies have reported that children over 12 years old have a strong desire to have genetic children, consistent with the present results (21). However, such results should be interpreted with caution as parental interventions cannot be ignored as a limitation of this study. In addition, in Q4 and Q5, a question was asked about the treatment being planned, and the proportion of those answering, “Know well” or “Know” increased with age. However, whether children understand correctly and how much detail they know has not been verified, so caution is also required regarding the interpretation of this result. Question 6 asked about the risk of impairing fertility during treatment of the underlying disease. This was a question that could be psychologically stressful to children. As expected, the ratio of “Know well” and “Know” was high and increased with age. This indicates the possibility that the content of information provided by doctors who treat primary diseases changes according to age. Similarly, a tendency was seen for an increasing understanding of the reason for visiting the FP hospital and knowledge about FP with increasing age. However, when asked if they would like to hear an explanation about FP, a certain proportion of respondents answered “Don’t mind”. In fact, some children may not have been positive about FP. As a post-explanation questionnaire, questions were first asked about the effect of treatment for the primary disease on fertility and the understanding of FP treatment. The survey with post-Q1 and -Q2 found no answers of “Don’t really understand” or “Don’t understand at all”, suggesting that basic understanding was relatively high in all age groups, showing the validity of providing explanations regarding FP. As in Q4 and Q5 above, this result has not been objectively confirmed. Therefore, one limitation is that the level of actual comprehension remains unclear. In addition, most children had not undergone objective evaluation of intelligence. Some diseases, such as brain tumors, cause developmental problems, and in this study only one patient with brain tumor (10 years old) had been tested for intelligence (Wechsler Intelligence Scale for Children, fourth edition). Intellectual development is an important factor that influences the significance of FP, and cannot be ignored, especially regarding FP for children. After the explanation of FP, willingness to receive FP was confirmed in post-Q3. Almost all children showed a willingness to undergo FP. The reason why children who were scheduled to undergo OTC also responded to OC was that the combined procedure was explained (post-Q4). In addition, since some respondents answered “Don’t really understand” regardless of age, tools to promote better understanding seem desirable. In any case, the majority of positive responses were attributed to the participation of the child in their own decision-making (21–23). In addition to pain and fear, cost concerns were raised as an issue for FP (post-Q5). Economic problems have been reported as a typical barrier for adults (24, 25) and this result is very important, since children can struggle with financial problems for FP in the same manner as adults. In addition, in post-Q6, -Q7, and -Q8, the lack of negative reactions to the explanations about FP indicates that direct explanation about FP to children is not harmful, regardless of whether they undergo FP. This was attributed to the participation of the child in the decision-making process, as described above. However, in post-Q8, some children (ages 6 and 11) said they did not really understand, indicating a need to develop even higher quality explanation methods. Finally, as mentioned above, the results of this survey cannot deny the influence of parental intervention. In other words, it is possible that the child gave the answer that the parent wanted. Of course, we asked the parents not to impose their opinions on the children, and basically only asked them to explain the questions. Therefore, it is presumed that the patient’s intention is basically reflected. However, it was not possible to exclude completely the involvement of parents due to issues of ethics and comprehension, but in the future, it would be desirable to plan a survey that children can complete by themselves using devices such as tablets using age-appropriate animation. Such developmental research will reveal actual children’s perceptions, understandings, and feelings. In addition, parental validation is important in research on FP for children. In terms of comprehension, perception, and attitude, I think we should also test our parents. Also, it is also necessary to collect more cases for boys. It is necessary to consider the differences between boys and girls in understanding and attitudes regarding fertility.

## 5 Conclusion

Categorically determining the lower age and intelligence limits at which explanations of FP can be understood is difficult. One challenge of pediatric FP is precisely the need to tailor explanations of procedures according to the understanding of the individual child.

## Data availability statement

The raw data supporting the conclusions of this article will be made available by the authors, without undue reservation. Please contact Nao Suzuki, nao@marianna-u.ac.jp.

## Ethics statement

The studies involving human participants were reviewed and approved by institutional review board at St. Marianna University School of Medicine. Written informed consent to participate in this study was provided by the participants’ legal guardian/next of kin. Written informed consent was obtained from the minor(s)’ legal guardian/next of kin for the publication of any potentially identifiable images or data included in this article.

## Author contributions

ST drafted the manuscript. ST, SF, MS, YY, TM, HK and NS designed the research and contributed to the critical discussion. ST, YI, YoS, HI, RK, ES, KI, YuS, KT, KOy, DK, KN, KOd, YH, LM, AI, AF, LA contributed to collecting and analyzing data. All authors contributed to the article and approved the submitted version.
